# Development and evaluation of a droplet digital PCR assay to detect *Brucella* in human whole blood

**DOI:** 10.1371/journal.pntd.0011367

**Published:** 2023-06-02

**Authors:** Jiayin Liu, Zhichun Song, Na Ta, Guozhong Tian, Xiaowen Yang, Hongyan Zhao, Dongri Piao, Yu Fan, Yu Zhang, Hai Jiang

**Affiliations:** 1 National Key Laboratory of Intelligent Tracing and Forecasting for Infectious Diseases, National Institute for Communicable Disease Control and Prevention, Chinese Center for Disease Control and Prevention, Beijing, China; 2 Wengniute Banner Center for Disease Control and Prevention, Chifeng, Inner Mongolia Autonomous Region, China; 3 Inner Mongolia Autonomous Region Comprehensive Center for Disease Control and Prevention, Hohhot, Inner Mongolia Autonomous Region, China; School of medicine, CHINA

## Abstract

**Background:**

With the development of domestic animal husbandry, the spread of brucellosis has accelerated, and the scope of the epidemic has expanded. The timely and accurate diagnosis of human brucellosis continues to challenge clinicians in endemic areas. Droplet digital PCR (ddPCR) technology can quickly and accurately determine DNA load in samples, providing laboratory evidence for diagnosis, prognosis and management of brucellosis patients. In this study, a ddPCR method was established to accurately quantify *Brucella* DNA load in whole blood samples, and its diagnostic, prognostic, and therapeutic value for human brucellosis was evaluated.

**Methods:**

Annealing temperature, primers, and probe targeting the *Brucella bcsp31* gene were optimised, and the sensitivity, specificity and repeatability of the ddPCR assay were assessed using 94 whole blood samples from 61 confirmed and 33 suspected cases. Results were compared with those of quantitative PCR (qPCR). Nine follow-up brucellosis patients were also analysed by the two methods after 2 and 6 months of treatment.

**Results:**

Optimal primer and probe concentrations were 800 nmol/L and 400 nmol/L, respectively, and the optimal annealing temperature was 55.3 °C. The ddPCR results showed that the limit of detection was 1.87 copies per reaction, with high repeatability. The positive rates for ddPCR and qPCR were 88.5% and 75.4% among 61 serum agglutination test (SAT) positive patients. In addition, 57.6% (19/33) of suspected sero-negative samples were positive by ddPCR, but only 36.3% (12/33) were positive by qPCR. Analysis of nine post-therapy follow-up brucellosis patients revealed that the Brucella DNA load in the whole blood samples decreased after 2 and 6 months of treatment, and was slightly increased following relapse and continuous exposure.

**Conclusion:**

The ddPCR assay showed good accuracy for whole blood samples, and could be a potential diagnostic and prognostic tool for detecting *Brucella*.

## Author summary

Brucellosis is an important zoonosis, it is still a global public health issue. Based on an evaluation from the World Health Organization (WHO), brucellosis cases have been reported in more than 170 countries, with ~500,000 new cases reported each year. During the past decade, outbreaks of human brucellosis have been reported in increasing numbers, and with an apparent geographic expansion from the historically affected north of China to southern provinces, due to increased movement of humans, animals, and animal food products from brucellosis-endemic regions. In recent years, advanced molecular detection methods have improved laboratory diagnosis, and could serve as important alternatives to culture and serological methods. In this study, a ddPCR method was established to accurately quantify *Brucella* DNA load in whole blood samples, and its diagnostic, prognostic, and therapeutic value for human brucellosis was evaluated. The ddPCR results showed that the limit of detection was 1.87 copies per reaction, with high repeatability. The positive rates for ddPCR was 88.5% among 61 SAT positive patients. In addition, 57.6% (19/33) of suspected sero-negative samples were positive by ddPCR. The ddPCR assay showed good accuracy for whole blood samples, and could be a potential diagnostic and prognostic tool for detecting *Brucella*.

## Introduction

Brucellosis is a neglected zoonosis and an important public health issue in developing countries. Humans are typically infected through contact with sick animals, especially goats, sheep and cattle, and through consumption of contaminated milk and milk products [1,2]. During the past decade, more and more outbreaks of human brucellosis have been reported, with an apparent geographic expansion from the historically affected North China to southern provinces, which is caused by increased movement of humans, animals, and animal food products from brucellosis-endemic regions [3].

Particularly in 2021, there was an overall resurgence of brucellosis; the annual number of cases reported nationwide China’s National Notifiable Disease Reporting System (NNDRS) was 69,767 (4.95/100,000). Compared with 2020, the number of reported cases and incidence were markedly increased by 47.67% and 47.05%, respectively. Based on an evaluation from the World Health Organization (WHO), brucellosis cases have been reported in more than 170 countries, with 500,000 new cases reported each year. However, the actual number of brucellosis patients is possibly 10–25 times the number of reported cases, which is largely due to misdiagnosis and underdiagnosis, especially in endemic areas [4].

The timely and accurate diagnosis of human brucellosis continues to challenge clinicians because of its non-specific clinical features, slow growth rate in blood cultures, and the complexity of its serodiagnosis [5–7]. Due to the challenges of culture and serological methods, advanced molecular detection methods have improved laboratory diagnosis, and could serve as important alternatives to culture and serological methods. Real-time PCR can be particularly useful in patients with specific complications, treatment failure and relaps [8–12].

Droplet digital PCR (ddPCR) is a new PCR technique, experimental procedure involves microdropping of the sample prior to conventional PCR amplification, whereby the reaction system containing nucleic acid molecules is divided into thousands of nano-droplets, each containing either no gene to be detected or one gene. After PCR amplification, each microdroplet is tested one by one, and the microdroplet with fluorescence signal is interpreted as 1, while the microdroplet without fluorescence signal is interpreted as 0. The starting copy number or concentration of the gene to be detected can be obtained according to the Poisson distribution principle and the number and proportion of positive microdroplets, without making a standard curve and without relying on the amplification Efficiency [13–15]. It performs excellently for absolute quantification of low-level targets and benefits from more robust resistance to various inhibitors than traditional real-time PCR, making it ideal for the detection of very low amounts of pathogen DNA in Whole blood. However, few studies on detecting *Brucella* in human whole blood by ddPCR have been reported. This work assessed the diagnostic performance of ddPCR, and evaluated its potential role in the prognosis and management of brucellosis patients.

## Materials and methods

### Ethics statement and whole blood samples

This study included 94 subjects, all with clinical symptoms of brucellosis and epidemiological risk factors. They were admitted to the Wengniute Banner Center for Disease Control and Prevention in Chifeng City, Inner Mongolia, from December 2021 to September 2022. Fasting venous blood (4 mL) was collected for brucellosisserological testing, and diagnosis of brucellosis was performed using the Diagnostic Criteria for Brucellosis WS269-2019 [16]. Suspected cases of brucellosis were defined as people with clinical symptoms (fever ≥37.5 °C, fatigue, night sweats and joint pain) and epidemiological risk factors for infection. Confirmed cases were defined as suspected cases with an antibody titre of ≥1:100 (++) in SAT or positive *Brucella* isolates. The ethics committee of the Inner Mongolia Autonomous CDC approved the study.

Written informed consent was been obtained from patients in accordance with the Declaration of Helsinki. We confirmed that identification information for all participants (including patient names, ID numbers, home addresses and telephone numbers) would not be included in recordings, written descriptions, or publications.

### Bacterial culture and DNA extraction

All bacteria strains used in the study were preserved in the laboratory of the National Institute for Communicable Disease Control and Prevention, Chinese Center for Disease Control and Prevention (Beijing, China). All experiments involving bacterial culture were approved and carried out in the laboratory under qualified biosafety conditions. *Brucella* reference strains were used in this study, including *B*.. *melitensis* bv. 1, 2 and 3, *B*. *abortus* bv. 1, 2 and 3, *B*. *suis* bv. 1 and 3, *B*. *canis*, and *B*. *Ovis* (Table 1). DNA from these strains and 94 whole blood samples was extracted using a QIAamp DNA Mini Kit (Qiagen 51304, Germany). A NanoDrop One instrument (Thermo Fisher Scientific, USA) was used to determine the DNA concentration.

**Table 1:** Source of reference strains.

Reference Strain	Source
*B*. *melitensis* bv. 1, 2 and 3	Department of brucellosis, ICDC, China CDC
*B*. *abortus bv*. 1, 2 *and* 3	
*B*. *suis bv*. 1 and 3	
*B*. *canis*	
*B*. *ovis*	
*Yersinia enterocolitica* O:9	Department of Diarrheal Diseases, ICDC, China CDC

### Primers and probes

We designed primers and probes according to *bcsp31* of *Brucella* as the target gene. Table 2 shows the list of primers and probes used in the study. All primers and probes were synthesised by Sangon Biotech Company (Shanghai, China).

**Table 2:** Sequence of primers and probe in this study.

Bacteria	Target gene	Sequence 5’-3’
*Brucella*	*bcsp31*	F:GCTCGGTTGCCAATATCAATGC R:GGGTAAAGCGTCGCCAGAAG P:FAM - AAATCTTCCACCTTGCCCTTGCCATCA- BHQ1

### Real-time PCR

The PCR mixture comprised 10 μL qPCR Probe Master Mix, 400 nM primers, 200 nM probes (Sangon Biotech, Shanghai), 1 μL sample DNA, and DNAase-free water to a final volume of 20 μL. Thermal cycling was performed for 3 min at 95°C to activate the enzyme, followed by 45 cycles of denaturation at 95 ° C for 15 s and annealing at 61 ° C for 30 s (with fluorescence measurement) [17].

### Droplet digital PCR

Primer/probe concentrations and annealing temperature were optimised. The ddPCR reaction mixture consisted of 10 μL 1 × ddPCR Supermix for probes (BioRad, City, USA), 800 nM specific primers, 250 nM probe, 2.0 μL DNA, and DNAase-free water to a final volume of 20 μL. The ddPCR assay was performed using a Bio-Rad ddPCR system consisting of a droplet generator and an automated reader.

Each ddPCR mixture was transferred to the droplet generator, generating up to 20,000 nanolitre-sized droplets, then loaded into a Thermal Cycler (Bio-Rad) for amplification. Thermal cycling was performed for 10 min at 95°C, followed by 40 cycles of denaturation at 95°C for 30s and 1 min annealing/extension from 50–62°C, then enzyme deactivation at 98°C for 10 min, and a final step at 12°C for at least 30 min to stabilise droplets. After amplification, droplets were measured using the reader, and data were analysed using Bio-Rad QuantaSoft Analysis Pro software.

### Analytical sensitivity and repeatability

*Brucella meilitensis* 16M DNA was serially diluted ten-fold to concentrations ranging from 38.7 ng/μL to 3.87 fg/μL, and dilutions were used to determine the sensitivity of ddPCR and qPCR assays. Each dilution was tested in triplicate. The linear relationship and limit of detection were determined. Repeatability was also evaluated according to the intra-batch and inter-batch coefficient of variation, respectively.

### Analytical specificity

To determine the specificity of the established ddPCR method, important serologically cross-reactive pathogens were detected, including *Yersinia enterocolitica* O:9, *Salmonella typhimurium*, *Vibrio parahaemolyticus*, *Escherichia coli* O157 and *Vibrio cholerae* O1(Table 1).

### Detection of whole blood samples and data analysis

To evaluate the efficiency of the established ddPCR assay for whole blood samples, 94 whole blood samples (61 SAT positive and 33 SAT negative) were tested by qPCR and ddPCR. For qPCR, any sample that had a cycling threshold (Ct) value <40 was considered positive, while any sample with a Ct value >40 was considered negative. Data were analysed using Microsoft Excel software and IBM SPSS statistics 21.0.

## Results

### Optimal thermal gradient for ddPCR

#### Determination of optimum annealing temperature

**Fig 1:**
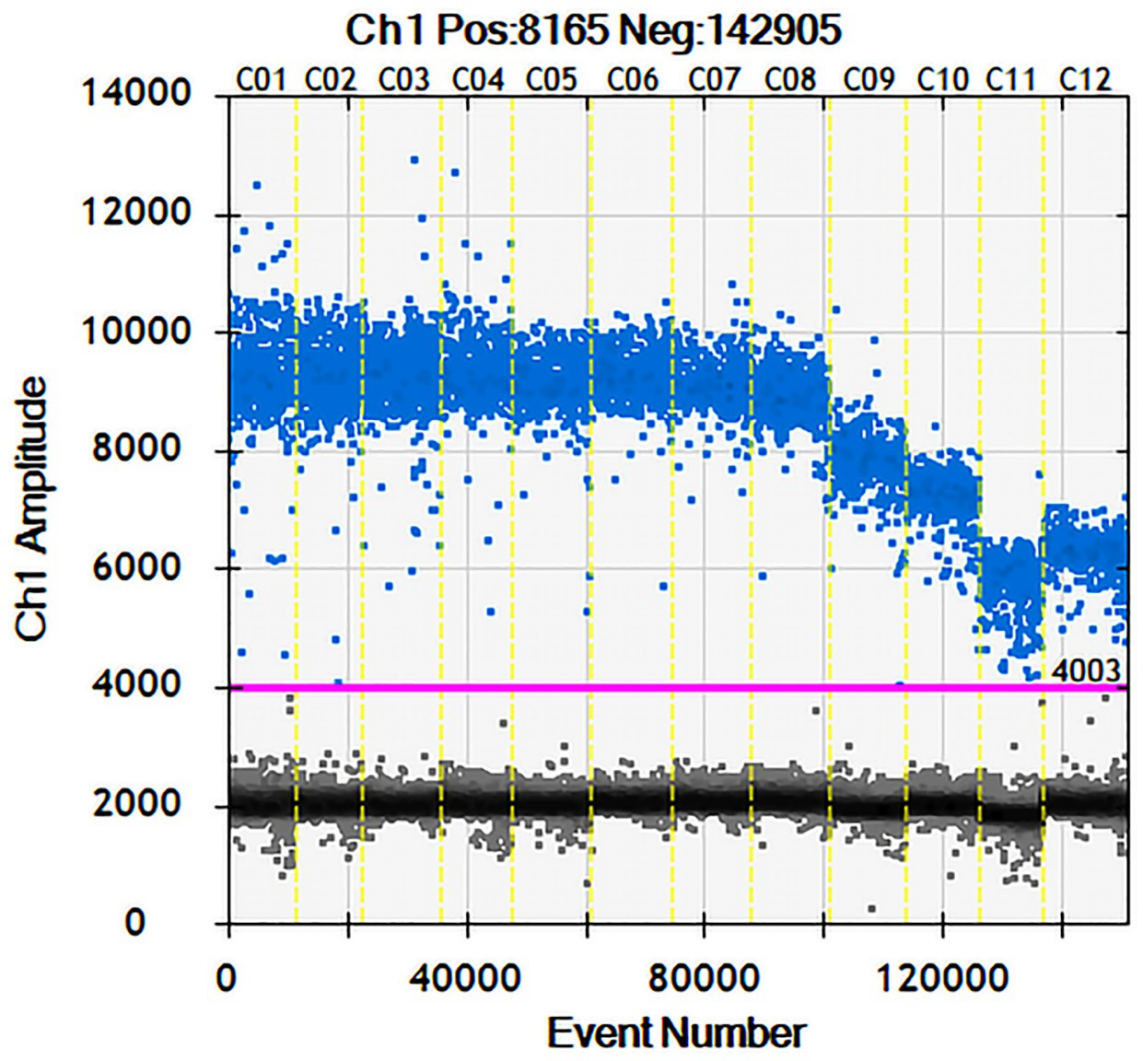
Scatter diagram of optimal annealing temperature in ddPCR. Note: the annealing temperatures of C01-C12 are 50.0°C, 50.6°C, 51.5°C, 52.7°C, 53.9°C,55.3°C, 56.7°C, 58.1°C, 59.3°C, 60.5°C, 61.4°C and 62.0°C respectively.

The greatest difference between the fluorescence values of positive (blue) and negative (grey) samples with the largest number of amplicons (positive droplets) was obtained at 55.3 ° C, hence this was selected as the optimal annealing temperature (Fig 1).

#### Determination of optimal primer and probe concentration

**Fig 2:**
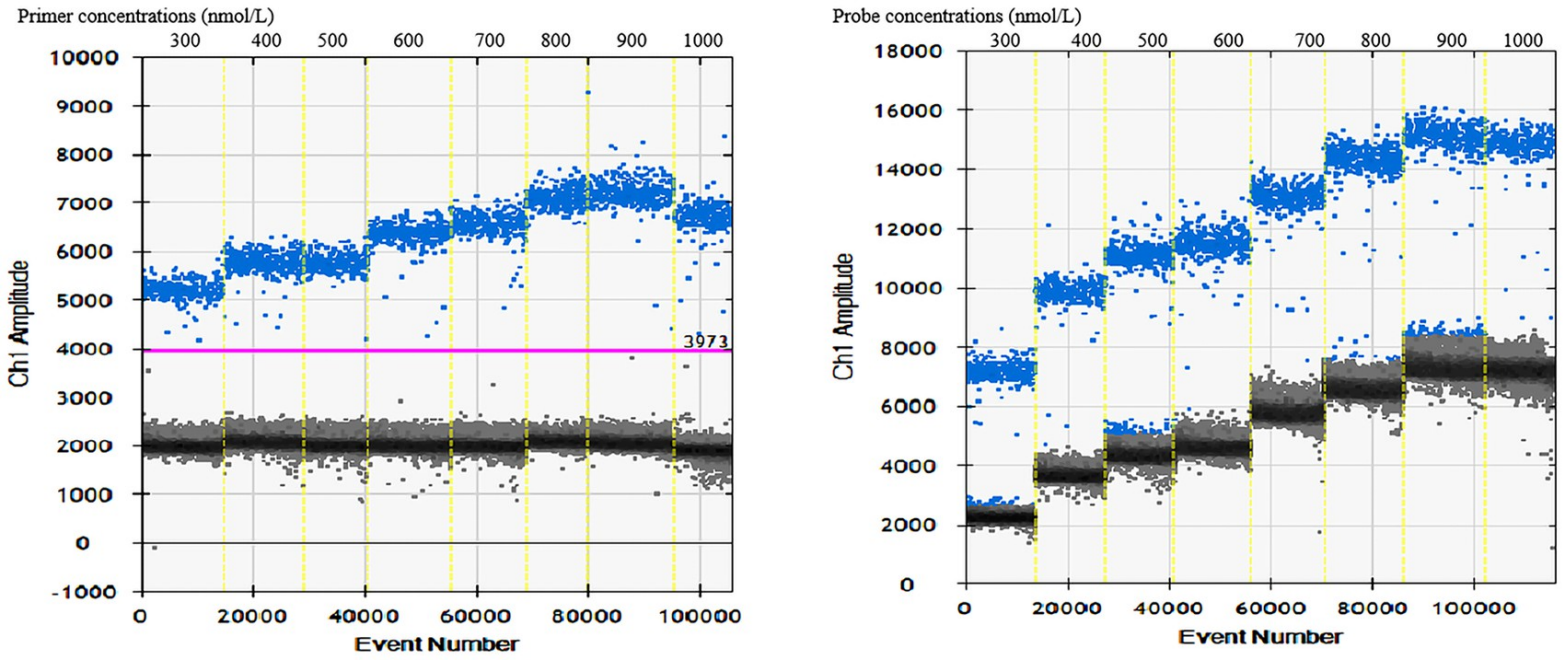
Scatter diagram of optimal primer and probe concentration. A: Determination of primer concentration: fixing the probe concentration to 250 nmol/L, and each primer concentration has been marked in the figure. B: Determination of probe concentration: fixing the primer concentration to 800 nmol/L, and each probe concentration has been marked in the figure.

The fixed probe was 250nmol/L, the primer concentration was changed, and the primer concentration was and the primer concentration was finally determined to be 800nmol/L (Fig 2A). Based on the comprehensive of aggregation, "tailing" phenomenon and amplification effect, the fixed primer was 800 nmol/L, and the probe concentration was determined to be 400 nmol/L by changing the probe concentration (Fig 2B).

#### Analytical sensitivity and repeatability of the ddPCR assay

**Fig 3:**
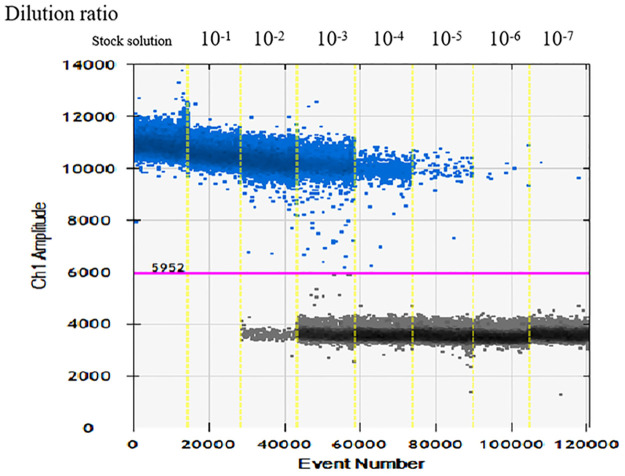
Gradient dilution amplification diagram of ddPCR *B*. *melitensis*16M DNA was sequentially diluted 10 fold from stock solution to 10^−7^.

The serially diluted DNA demonstrated good PCR efficiency in both qPCR and ddPCR assays. In qPCR, the standard curve exhibited good linearity (*y* = -3.2607x + 44.136) with a correlation coefficient R^2^ = 0.998. The limit of detection was 3.87 fg/μL, and concentrations below this were not detectable (Fig 3). In ddPCR, the limit of detection was 1.87 copies per reaction. To assess repeatability, the intra-batch and inter-batch variation coefficients were calculated (Table 3), and the results revealed good repeatability for detection of *Brucella*.

**Table 3:** Results of ddPCR repeatability test.

		Intra-assay variation			Inter-assay variation		
Dilution Numbering	Concentrations (/μL)	Mean (copies/reaction)	SD	CV	Mean (copies/reaction)	SD	CV
1	38.7ng	2.00×10^7^	0	0	2.00×10^7^	0	0
2	3.87ng	2.00×10^7^	0	0	2.00×10^7^	0	0
3	387pg	1.074×10^5^	1708.8	0.016	1.076×10^5^	3103.0	0.029
4	38.7pg	11046.67	280.2	0.025	10744.4	282.6	0.026
5	3.87pg	1075.33	35.6	0.033	1029.6	50.1	0.049
6	387fg	91.33	8.1	0.088	91.6	1.0	0.01
7	38.7fg	9.47	2.3	0.24	9.5	1.6	0.17
8	3.87fg	1.87	0.50	0.27	2	0.4	0.20

#### Analytical specificity of the ddPCR assay

**Fig 4:**
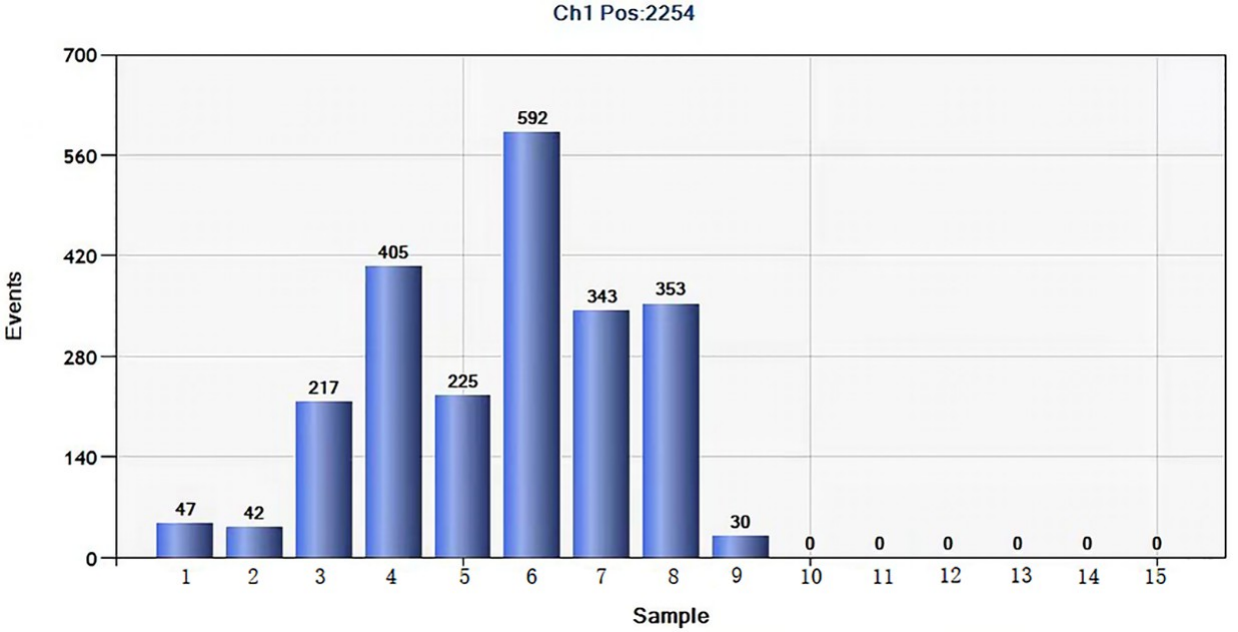
Results of ddPCR specificity test. Note: 1–9 *B*. *melitensis* bv1, 2 and 3, *B*. *abortus* bv1, 2 and 3, *B*. *suis* bv1, *B*. *canis* and *B*. *ovi*s. 10–15 specific strains: *Yersinia enterocolitica* O:9, *Yersinia enterocolitica* O:3, *Salmonella typhimurium*, *Escherichia coli* O157, *Vibrio parahaemolyticus*; *Vibrio cholerae* O1.

For specificity analysis, DNA from *Y*. *enterocolitica* O:9, *S*. *typhimurium*, *V*. *parahaemolyticus*, *E*. *coli* O157 and *V*. *cholera*e O1 were tested by ddPCR assay. As shown in Fig 4, only *Brucella* DNA tested positive, while DNA from other organisms tested negative. The results confirmed that this method was specific for detection of *Brucella*.

#### Evaluating the ddPCR assay using whole blood samples

Of 61 confirmed sero-positive SAT samples analysed by ddPCR and qPCR, 54 and 46 were positive, respectively. Of 33 suspected sero-negative SAT samples analysed by ddPCR and qPCR, 19 and 12 were positive, respectively. As shown in Table 4, the positivity rate of ddPCR (88.5%) was higher than that of qPCR (75.4%).

**Table 4:** Whole blood samples detected by qPCR and dd PCR.

	ddPCR		
qPCR	Positive	Negative	Total
Positive	45	3	48
Negative	9	4	13
Total	54	7	61
*χ*^*2*^ = 4.08		*P<0*.*05*	

#### Prognostic and therapeutic value of ddPCR for brucellosis detection in follow-up patients

Whole blood samples were drawn from nine confirmed patients at the time of diagnosis, and after 2 and 6 months of follow-up treatment. Table 5 shows changes in DNA load during the treatment. At diagnosis, ddPCR gave positive results for all nine infected patients, with a mean bacterial load of 22.2 copies/reaction (range 3 to 92 copies/reaction). After concluding treatment (2 months), the bacterial load fell slightly, with a mean bacterial load of 8.4 copies/reaction (range 2.2 to 15.2 copies/reaction). However, the mean bacterial load was 5.2 copies/reaction (range 0 to 12.6 copies/reaction) after 6 months. Furthermore, an increase in copies was only observed for cases 8 and 9 after 6 months of follow-up treatment.

**Table 5:** Changes of Ct values and copies after follow-up treatment.

	On site		2th month		6th month	
Sample	Ct	Copies/reactions	Ct	Copies/reactions	Ct	Copies/reactions
1	33.32	30	35.87	11	37.54	2
2	33.67	16	34.88	12.2	36.62	4.6
3	34.71	12.2	34.73	8.2	35.91	4.2
4	32.75	92	34.24	10.2	34.39	3
5	34.75	18	35.63	7.4	34.98	2.6
6	34.03	6.6	35.76	2.2	37.8	0
7	35.27	7.8	33.95	6	34.57	7
8	35.07	14.2	36.53	3.6	35.32	10.4
9	36.47	3.0	34.25	15.2	34.54	12.6
x±S	34.45±1.13	22.2±27.3	35.09±0.89	8.4±4.2	35.74±1.31	5.2±4.1

## Discussion

At present, the commonest methods for detection of brucellosis are isolation of *Brucella*, and serological and molecular biology techniques. The gold standard for brucellosis is isolation of *Brucella*, which is not suitable for routine clinical practice due to the low clinical isolation rate, time-consuming nature (more than 3 days), and risk of laboratory-acquired infection [18,19]. SAT approach is a serological method for brucellosis confirmation. However, operation is troublesome and the results require subjective judgment, which is prone to false negative or false positive results. Real-time PCR is currently the most-used molecular method in the diagnosis of human brucellosis. Real-time PCR is a rapid diagnostic tool for detection of *Brucella spp*. DNA in human serum samples [20–22]. Evolution of *Brucella melitensi*s DNA load during therapy and post-therapy follow-up in human whole blood samples by real-time PCR demonstrated its potential value for management of brucellosis patients [23]. Another study assessed whether a bacterial load cutoff can differentiate patients with active brucellosis from those with a cured past infection [24]. Further studies are needed to determine the true meaning of the transitory appearance of low-grade bacterial load in patients with a history of disease [25]. ddPCR technology, an improvement on traditional PCR, is based on limiting dilutions and Poisson statistics. Indeed, ddPCR has emerged as a highly sensitive and precise method for nucleic acid quantitation of clinical samples. One ddPCR assay for detection of Bovine Herpesvirus 1(BoHV-1)DNA in semen samples achieved good accuracy for mixed samples, and could provide a diagnostic tool for detecting BoHV-1 [26]. Because it is more sensitive than qPCR, ddPCR can detect target DNA at low concentrations. Therefore, this method has been used to diagnose a variety of diseases, and is especially useful for low bacterial loads, allowing more timely treatment and prevention of bloodstream infections (BSI). The feasibility of detecting pathogen DNA in whole blood using the ddPCR technique and the diagnostic accuracy of ddPCR to detect BSI were explored by Wouters et al. [27]. The clinical application of mNGS-based and ddPCR-based methods for rapid and accurate detection of pathogens in BSI was comprehensively evaluated by Hu et al.. The ddPCR method is more useful for rapid detection of common isolated pathogens in critically ill patients with suspected BSI [28]. A multiplex ddPCR method was established for simultaneously detecting five high-risk bacterial biothreats, and the more sensitive than qPCR, suggesting that ddPCR may be potentially suitable for rapidly detecting or screening the five selected bacterial biothreats in suspicious soil samples [29]. Tian et al. developed and optimized a ddPCR method for detection of *Brucella* in blood specimens from an outbreak. Samples from 18 staff and 8 sick sheep had 16 and 5 positive respectively by ddPCR. This study showed that ddPCR was significantly more sensitive than bacterial culture [30]. Mei et al. constructed a *Brucella* microdrop digital PCR method. 136 bovine vaginal swab samples were detected by the bacterial culture and the ddPCR methods. The results proved that 78 positive samples and 58 negative samples were detected by both methods, and the compliance rate of both methods was 100% with high specificity [31]. In addition, the ddPCR assay yielded significantly fewer false negative and false positive results than qPCR for samples with low bacterial load. In the present study, whole blood samples of 61 SAT positive patients were detected by ddPCR and qPCR, and positive rates were 88.5% and 75.4%, respectively, which proved that ddPCR was more sensitive. Additionally, 33 SAT negative samples were analysed by the two methods, and 19 and 12 were positive, respectively. Because there is a narrow window for antibody detection, continuous and regular detection of antibody-negative suspected cases should be carried out. With the prevalence of COVID-19, county-level CDCs in China now have high qPCR detection capability. Therefore, we suggest that brucellosis high-risk populations should be screened using serological-based and molecular-based methods, which can reduce the missed diagnosis rate.

When nine post-therapy follow-up patients were observed, we found that DNA copies in whole blood samples were decreased at different treatment stages. Table 4 shows that bacterial load was higher in the early stages of disease compared with the recovery stage, and a higher bacterial load may indicate disease progression, suggesting that accurate quantification via ddPCR at different timepoints may be key to monitoring the course of disease and evaluating responses to therapy. Based on clinical information, case 1-case 7 showed a low bacterial load at the end of the 2 months course of treatment, and there was long-term improvement in symptoms. However, DNA load for case 8 and case 9 showed a slight increase during treatment for 2 and 6 weeks. Based on epidemiological information, the two patients were from rural areas, and often came into contact with cattle and sheep, which may lead to relapse and repeated infection during treatment. These findings suggest that when upon relapse and continuous exposure to *Brucella* during treatment, patients can experience DNAemia over a prolonged period. Further studies are needed to determine the true meaning of the transitory appearance of low-grade bacterial load in follow-up patients with a history of disease [25].

ddPCR is advantageous for detecting low quantities of circulating DNA. *Brucella* is facultative intracellular pathogens, and the bacterial concentration in the blood of patients with brucellosis is usually low. Serodiagnostic tests lack specificity in patients suffering relapse. Therefore, ddPCR has great potential for use in the follow-up of brucellosis patients after completion of antibiotic treatment, and for early detection of relapses. However, the accuracy of data interpretation must be improved to enable the use of this technology in clinical settings.

This study has some limitations. First, blood culture could not be performed due to a relatively low positive rate and poor pathogen detection capacity in county-level CDCs. Second, our study included a relatively small sample sizes. Hence the results need to be further verified by expanding the sample size, especially the number of follow-up patients.

## Conclusion

The established ddPCR assay displayed good accuracy, repeatability and specificity. It could be used for accurate detection of *Brucella* in human whole blood samples. Therefore, it could provide a potential diagnostic and prognostic tool for diagnosis and management of brucellosis patients.
